# Donor notification of permanent deferral: a qualitative study on the perceptions and practices of notifier and blood donor in Mexico

**DOI:** 10.1186/s12913-022-08103-1

**Published:** 2022-06-10

**Authors:** V Moisés Serrano-Delgado, Edith Valdez-Martínez, Horacio Márquez-González

**Affiliations:** 1 Central Blood Bank of the National Medical Centre ‘La Raza’, Mexican Institute of Social Security, Mexico City, Mexico; 2 Health Research Council of the Mexican Institute of Social Security, Mexico City, Mexico; 3 Department of Clinical Research, Children Hospital of Mexico ‘Federico Gomez’, Mexico City, Mexico

**Keywords:** Blood donors, Permanent deferrals, Health personnel, Notification process, Qualitative study, Mexico

## Abstract

**Background:**

Informing about permanent deferral requires a process that links the notifier with the donor in a particular way. Little is known about the type of information and how it is disclosed to the donors. The current study aimed to examine perceptions and practices of notifier and blood donor within the framework of the notification process of permanent deferral and from the perspective of the notifier―blood donor relationship.

**Methods:**

A qualitative study with in-depth interviews. The participants were 13 notifiers and 25 permanently deferred donors. Participants were recruited from a national blood bank and a state’s blood bank. The entire dataset/narratives were analysed using the method of thematic analysis.

**Results:**

The disclosure of permanent deferral was understood as a matter of disclosing the serological test results and their medical meaning along with a concise explanation of the deferral status with regard to future blood donation and the plan to be followed. The notifiers preferred to act in accordance with the standard protocol despite acknowledging the adverse psychological and social effects to which donors are exposed when they are informed of the possible disease and the consequent permanent deferral. Donors described a variety of psychological and social affectations. They valued honesty in the communication, the clarity of the information provided and a greater involvement of the notifier.

**Conclusion:**

Even though the notification process does not imply that medical care is being offered to donors, the notifier is the administrator of the well-being of the donor. Notification must not be considered as something apart from care, since it is intimately related to the health of each of the donors and their medical care.

**Supplementary Information:**

The online version contains supplementary material available at 10.1186/s12913-022-08103-1.

## Background

### What constitutes best practice for notifying permanent deferral?

Current international and national regulatory guidances [1–3] define notification as the information provided to donors about reactive or positive results of screening for serological markers of infectious diseases and the subsequent permanent deferral as donors. The international guidance [1–3] requires that the notification be made during a counselling meeting. The *Food and Drug Administration* (FDA) [1], in particular, advises that (a) the notification should last six minutes, (b) it should not be part of the practice of medicine and (c) the notification process should include a minimum of three attempts to notify the donor within eight weeks of determining that the donor is deferred.

The international guidance [1, 2] also states that blood banks (BBs) have the flexibility to choose the manner in which they notify donors and that the notification should be by a physician or any other person medically qualified. The documents do not define the meaning of ‘a person medically qualified.’ These standard procedures concur in stating that the deferred blood donor must be the person to be notified. European Guidelines (*Guide to the Preparation, Use and Quality Assurance of Blood Components Recommendations*) [2] indicate that notifications to a partner or partners must be voluntary; notwithstanding, the donor has the ethical obligation to inform a sexual partner, and the BB must encourage and support them to do it.

### Context

#### Demographic aspects of blood banks (BBs) in Mexico

BBs in Mexico are regulated by the National Centre of Blood Transfusion [4] and the Federal Commission for Protection Against Sanitary Risks [5]. The former focuses its objectives on achieving self-sufficiency, security, quality and the rational therapeutic use of blood units. The latter is the organisation in charge of the issuance of sanitary licences and carrying out of the verification of the functioning of BBs and blood transfusion services so that they comply with Mexican official standards [3].

The Mexican health system has two sectors: public and private. The public sector includes the health institutions belonging to the social security system and the institutions and programmes that provide medical care to the population without social security. Within this sector, there are 558 BBs, 4,511 transfusion services and 222 blood stations [6]. Of the 558 BBs, 40% belong to the private sector and 60% to the public sector [6]. Briefly, a BB is a place where blood gathered as a result of blood donation is analysed, stored, and distributed, for later use in blood transfusion. A BB also receives the blood units from blood stations, and it is the only one authorised to inform blood donors of their laboratory test results. A blood station is an authorised place to recruit donors, to draw blood, to store it temporarily, and to transport it to a BB. A transfusion service is a place that receives the blood units for their ultimate clinical use.

The Pan American Health Organisation [7] states that in the information reported by 36 countries of Latin America and Caribbean, for the years 2016 and 2017, Brazil has the biggest percentage (38.3%) of blood donated, followed immediately by Mexico (22.7%). As for Mexico, of these donations, 97% occur following the family/replacement donation strategy in which health professionals ask relatives to donate blood for a hospitalised patient in the institutions where he/she is being provided care. This explains why only 3% of the donors are voluntary.

#### National epidemiologic aspects of the deferral of blood donors

Although there are no trustworthy and available national statistics that permit the determination of the frequency of each of the causes of deferral before and after donation, the numbers presented in this section are those recorded in the electronic database for the registry of blood donors (known as HEXA-BANK, unpublished data) of the Central Blood Bank of the National Medical Centre ‘La Raza’ of the Mexican Institute of Social Security. This database is accessed from an internal computer system and is officially recognized by the Mexican system for BB accreditation.

The HEXA-BANK database shows that, in Mexico, from 1 January 2015 to 31 December 2019, the causes for deferral pre- and post-donation are as follows:

*Before donation*. The most frequent causes of temporary deferral are low levels of haematocrit/haemoglobin (25%), lipemia (15%), elevated leukocyte counts (10%) and high levels of haematocrit/haemoglobin (9%). The principal causes of permanent deferral are seizures, systemic arterial hypertension and neoplasms. These events jointly represent <1% of the total of deferrals.

*After donation*. The main causes of permanent deferral are reactive serology test for infections with *Treponema pallidum*, *Trypanosoma cruzi* (*T. cruzi*), hepatitis B virus (HBV), hepatitis C virus (HCV), and human immunodeficiency virus (HIV).

Of the 500,473 blood donors registered, a total of 3,961 (0.79%) were deferred permanently as they presented reactive tests (serology with immunoassay and nucleic acid test) for infection with HBV, HCV and HIV. For these 3,961 donors with reactive tests, the frequency distribution by virus type is as follows: HBV, 13% (*n* = 516); HCV, 47% (*n* = 1,860) and HIV, 40% (*n* = 1,585). In contrast, of the 500,473 donors, only 602 (0.12%) were confirmed as positive (by amplification of nucleic acids) for infection. The frequency distribution by virus type, for this group, is as follows: HBV, 23% (*n* = 137); HCV, 45% (*n* = 269) and HIV, 33% (*n* = 196). The fall-off between immunoassay results and nucleic acid test may represent past infection with viral clearance or initial false positives [8].

Furthermore, a total of 2,439 (0.49%) donors were permanently deferred as they presented a serology reactive to immunoassay *via* chemiluminescence for *T. cruzi* (51%, *n* = 1,253) and *Treponema pallidum* (49%, *n* = 1,186). Of the 1,253 donors with serology reactive for infection with *T. cruzi*, only 74 (6%) were confirmed as positive (*via* immunofluorescence).

#### The notification process in Mexico

There are two events in the notification process: the first occurs when the donor is contacted through phone call, postcard, email, telegram, or visit in his/her domicile or at his/her place of work. This part of the process is carried out by non-medical personnel. The second event occurs when the donor comes to receive the information about his/her screening test results and meets face-to-face with the notifier (a professional appointed by the BB, in practice, this is usually a physician). Positive or indeterminate or reactive cases are referred to a medical attention unit for epidemiologic follow-up and medical treatment as appropriate. These positive or indeterminate or reactive cases are labelled and entered in the database of the BB as not eligible for future blood donations; that is, they are entered in a manner that blocks their reregistration. This restriction is kept in place even when subsequent results are reported as negative [3].

### Why is this study important?

At present, medical care is inextricably tied to blood and its products or components. Blood donors are, therefore, central agents. The notification of permanent deferral (because of a possible disease) involves a process that binds the notifier with the donor in a particular manner. This implies that the notifier is, by necessity, the *de facto* administrator of the well-being of the deferred donor. The well-being of the blood donor will be a determining factor of the manner in which the *de facto* administrator understands the nature and purpose of notification work.

The obligation to inform donors of the results of the serologic tests is considered to be a basic topic in the process of notification. When the disclosure of the information is inadequate, donors find themselves at a disadvantage to elaborate rational choices; *i.e*., to elaborate cognitive responses related to accepting or rejecting the notifier’s recommendations. Quantitative studies have estimated the magnitude of the consequences of this event. For example, they report that deferred donors suffer from varying levels of emotional responses and confusion while being left with unanswered questions after being notified [9, 10]. The results of a qualitative study [11] that explores, through interviews, the experience of permanent deferral from the perspective of 28 donors points in the same direction. The authors of the mentioned study show that the participating donors present a variety of negative emotional and behavioural answers (e.g., confusion, shock, disbelief, panic, fear, anger, stigmatization, and loss).

In a 2018 literature review [8] related to the notification of ‘false-positive’ results (of the serologic screening tests) to the blood donors, the authors emphasise the importance of acknowledging the psychological damage caused by the stress and anxiety that the donors feel during and after the notification. Another 2020 literature review [12] about the frequency and implications of indeterminate screening results of blood donors contends that a donor who is informed of this type of results becomes alarmed and angry. It also indicates that the indeterminate results of the screening tests are rarely associated with specific risk factors and that there are cases in which the uncertainty will never have a satisfactory resolution.

To the best of our knowledge, this study is the first to explore the information processing [13] of the notification process even though it is a topic in which important problems occur. For example, the selective perceptions of the notifier and the donor can attach a special meaning, to certain words, that implies prejudices and biases that distort the processing of the information. Thus, the type of information and the way the notifier communicates the screening test results to the donor will impact the donor’s emotions and the decision that he/she makes; for example, accepting or rejecting to undergo new serologic tests, to visit his/her physician, to attend epidemiological follow-up, and to be treated as appropriate, among other procedures. The present study is, therefore, necessary and opportune because understanding the reality of the process of notification (through the examination of the perceptions and practices of the participating agents) as it occurs naturally fills up a gap in the knowledge of this unique clinical relation.

The objective of this study was to examine perceptions and practices of notifier and blood donor within the framework of the notification process of permanent deferral and from the perspective of the notifier―blood donor relationship.

## Methods

### Design and setting of the study

From January 2014 to December 2015, a qualitative study was carried out which included individual, face-to-face, semi-structured, in-depth interviews with notifiers and donors. This qualitative research method is particularly well suited for understanding personal perceptions, practices, experiences, and contextual circumstances [14].

The interviews were conducted in two BBs: the Central Blood Bank of the National Medical Centre ‘La Raza’ of the Mexican Institute of Social Security and the Blood Bank of the State’s Blood Transfusion Centre of the State of Hidalgo. The first BB was selected as it recruits donors nationally and because the blood provenance is of familial/replacement type. The second was randomly selected from the States’ BBs belonging to the Secretariat of Health; it recruits donors from various States of the country.

### Characteristics of participants

The study included health professionals who notified donors of their deferral status. It also included donors that were classified as permanently deferred and that had been notified of their deferral status within the first 30 days of any of the following results of tests for communicable disease agents: *Treponema pallidum*, *T. cruzi*, HBV, HCV, and HIV.

Both notifiers and blood donors were recruited through the participating BBs.

#### Terminology

Here, the word ‘donor’ is used to refer to blood donors who as a result of having donated blood, because they were considered healthy, were later classified as permanently deferred. The terms ‘medical notifier’ and ‘non-medical notifier’ are used as follows: the first refers to the physician who interviews the donor to disclose screening test results and to prescribe the medical course to be followed. The second refers to the health professional who calls the donor to request him/her to come to the BB to find out the test results.

### Sample and sampling

The sample size was determined using the Sandelowski criterion [15]. The criterion states that in a qualitative study, the sample size is not determined *a priori* but is based on the robustness of the theory that is generated. A robust theory is one that is sufficiently deep and wide to be able to explain the knowledge generated. Following this criterion, the sampling was stopped when the data became repetitive or redundant, and new analysis only confirmed what had already been established.

Purposive sampling [16] was used. The idea was to deliberately select those informants who would provide a wealth of information for in-depth analysis. Hence, this study included an extensive sampling; that is, a sampling oriented toward finding variations in the characteristics of the informants and thereby enriching the information gathered. For example, for health professionals, age, gender, marital status, labour category and seniority in the BB were considered, whereas for the deferred blood donors, age, gender, marital status, occupation and schooling were considered. Donors were initially interviewed during the first 30 days after having been informed that they were classified as permanently deferred because of reactive serology for any of the following results of a test for communicable disease agents: *Treponema pallidum*, *T. cruzi*, HBV, HCV and HIV. Thus, the donors interviewed first were those who came to the BB to be notified of their laboratory results or who just left the notifier’s officine. Afterwards; following the extensive sampling strategy, a second sample of donors interviewed were those with reactive screening test that did not come to the BB for their results; they were identified from the physical (notebooks) and the electronic databases of the participating BBs.

### Data gathering

Before the beginning of the study, authorisation from the directors of the participating BBs was requested by means of a letter of invitation that explained the nature of the study along with a written memorandum from the pertinent research ethics committee. During this stage, the interviewers (three clinical psychologists and one physician) were trained in the technique of semi-structured, in-depth interviewing by (AZ) a skilled social scientist. When it was corroborated (by means of pilot tests) that they had mastered the technique for data gathering, the field study was initiated. To avoid possible psychologist/interviewer or physician/interviewer role conflict, the interviewers were never in charge of the psychological care or medical care of those interviewed.

An interview topic guide was developed (Additional file 1) based on theoretical knowledge and group discussions with the research team. This guide underwent adaptations throughout the study, as a function of the analysis of the information being generated and new threads of questioning being identified. The topic guide included issues related to values, beliefs, preferences, interests, anxieties, concerns, and ideas associated with permanent deferral. Further information such as demographic data was also collected during the interviews. Because unanticipated emotional issues could arise from these interviews, in cases of distress, interactions were guided by the respondents’ emotional needs.

The individual, face-to-face interviews were conducted in private (i.e. a researcher together with a psychologist and an interviewee, or two psychologists and an interviewee) at a site chosen by the interviewee. The interviews were audio-recorded, and an experienced medical transcriber (AL) transcribed all of the recordings verbatim. The interviewers independently reviewed each one of the recordings with their own respective transcriptions to corroborate the correct emphasis of each one of the arguments.

### Analysis

The entire dataset/narratives were analysed using the method of thematic analysis [17]. This method is particularly well suited for organising and summarising the findings from a large, diverse, and complex body of research.

In order to examine participating subjects’ perceptions, practices and experiences with the notification process of permanent deferral, an inductive thematic analysis [14, 18] was initially performed. The primary purpose of the inductive approach was to allow research findings to emerge from dominant or significant themes inherent in the raw data, without the restraints imposed by structured methodologies. Accordingly, the inductive thematic analysis involved constant, interactive and reflexive revisions of each one of the narratives, independently completed by the two researchers (MS and EV); any discrepancy was resolved by reaching a consensus. The researchers and research assistants (the psychologists) held various joint sessions throughout the study. The data collection and analysis stages in this study were undertaken concurrently, the previous stages of the process were reread before undertaking further analysis to ensure that the developing themes were grounded in the original data.

While inductive thematic analysis offered insights into the personal perceptions and practices about the notification process of permanent deferral, a deductive thematic analysis was performed subsequently to test whether data were consistent with assumptions, theories or hypothesis identified or constructed in other primary research studies.

The major themes that emerged from the analysis/interpretation of the narratives were defined and refined over the period of analysis.

The information generated by means of the interviews was captured for its analysis and managed by use of the software Atlas.ti version 8.0 for Windows (Cincom Systems, Inc., GmbH, Berlin).

## Results

### Demographics

The demographics and relevant characteristics of the participating notifiers (*n* = 13) and donors (*n* = 25) were quantitatively summarized and are showed in Table 1.

**Table 1:** Demographic characteristics of the informants

**Variable**		**Notifiers** **(*n* = 13)**	**Donors** **(*n* = 25)**
**Age in years (median, range)**		35 (28-58)	36 (24-60)
**Gender**	Female	9	18
	Male	4	7
**Marital status**	Married	8	11
	Single	3	8
	Living together	2	5
	Widow/widower	-	1
**Education**	Primary	-	2
	Secondary	-	9
	Preparatory	-	5
	Bachelor´s degree	5	8
	Speciality	8	1
**Religion**	Catholic	9	19
	Christian	-	4
	Atheist	1	2
	Missing data	3	-
**Occupation**	Medical specialist	8	1
	* Professional activities	5	4
	Businessman	-	4
	Builder	-	4
	Housewife	-	3
	Clerk	-	2
	Machine operator	-	2
	Security guard	-	2
	Photographer	-	1
	Maintenance worker	-	1
	Artisan	-	1
**Place of residence**	State of Mexico	-	10
	Mexico City	7	7
	Hidalgo	6	6
	Puebla	-	1
	Tlaxcala	-	1
**Diagnosis**	Syphilis	-	9
	Hepatitis C	-	8
	Chagas disease	-	3
	Indeterminate	-	3
	Human Immunodeficiency Virus	-	2
**Work seniority as notifiers (median, range).**		24 (1-300) months	-
**Time elapsed from the date of notification to the date of the interview (median, range).**		-	14 (1-1460) days
**Duration of the interviews (average standard deviation).**		65.5 (± 24) minutes	35.3 (± 13) minutes

^*^In the group of notifiers: generalist physician (*n*=1), psychologist (*n*=1), social worker (*n*=2) and accountant (*n*=1)

^*^In the group of donors: architect (*n*=1), lawyer (*n*=1), teacher (*n*=1), biotechnologist (*n*=1)

The synthesis of the narratives of the 13 notifiers revealed four themes: (a) relationship-centred notifier; (b) beliefs, emotions and feelings; (c) consequences of tagging the donor with the expectation of a disease and (d) barriers and facilitators. In the group of 25 donors, four themes emerged: (a) beliefs, emotions and feelings; (b) consequences of tagging the donor with the expectation of a disease; (c) idea of permanent deferral or ‘lock’ and (d) barriers and facilitators.

The abbreviated numeric expressions in brackets indicate the correct source of the excerpts throughout the narratives.

### Notifiers

#### Relationship-centred notifier

All notifiers concurred in stating that the information given to the donors was based on the results of the screening tests; several stated *“I devote whatever time is necessary to communicate the lab results with the intention of dealing with the doubts and anxieties of the donor and to make him/her aware of getting medical attention and treatment”* [P02, P03, P08, P09, P10, P25]. Likewise, many of the notifiers stated that they were aware of the fact that the disclosure of the screening test results for communicable disease agents puts the donor in a vulnerable situation [P01, P02, P03, P08, P09, P25, P29, P31, P37]. More than half also expressed concern about how much donors understand: *“Their emotional situation is such that it reduces their capacity to understand and discern”* [P01, P02, P25, P28, P29, P 31, P37].

That is the reason why they opt to be brief and punctual in the type and quantity of the information they disclose while centring on informing the donor about the importance of seeking medical care as appropriate: *“I tell him/her: ‘I will send this sheet to your general physician so that you will get the treatment’. And I add: ‘You can search on the Internet if you like’. I do not go farther, because they prove to be unwilling… Then I only give the information with the attitude if he/she wants to believe me, well, and if not, not”* [P01 (01:10)].

A notifier [P28] added that it is not his obligation to notify the sexual contacts of the donor: *“I cannot sit both down here to tell them…because it is not a counselling session, I am simply providing a reactive test result to a donor that came up”* [P28 (28:15)].

More than half of the notifiers stated that, because of legal issues, despite the diagnostic uncertainty in the initial screening test result, they feel obligated to inform the donors that they will be unable to donate blood in the future, even if the results of the diagnostic confirmation were negative [P03, P09, P25, P30, P31, P37, P38].

*“We say: ‘It definitely cannot be done because of the law’; that is, we blame the law. We tell them: ‘You will not be able to donate; the law does not allow it’. And they are told, as to give them some hope: ‘You know what? Laws change and we do not know if in the future the law will permit you to donate blood…Right now you are a permanently deferred donor, yet the term definitive or permanent does not exist, remember that it is possible that the law will change’”* [P25 (25:84)].

Many of the notifiers recognised that the notifier―donor relationship is not among peers. They mentioned: *“We limit ourselves to acting in adherence to the Mexican Official Standard for the Management of Human Blood”* [P01, P09, P28, P29, P30, P31, P37, P38]*.* They also explained that as notifiers, they protect their integrity and reduce the possibility of grievances among the donors: *“Being sued and other claims because of moral damage to the donor, who upon repeating the lab tests finds that his/her results are negative”* [P08, P10, P31, P38].

#### Beliefs, emotions and feelings

More than half of the notifiers affirmed that *“The blood donors always come expecting to have a possible disease, this causes them to experience a multitude of emotions, and they identify it as something difficult that changes their lives; they go away feeling pressured and without understanding the information they have received in reference to the lab results”* [P01, P02, P03, P08, P30, P31, P37].

There were some who believed that this *“multitude of emotions”* is influenced by *“the life history of the donor”*, “*the type of information disclosed”*, *“the relevance that is given by the notifier to the risk factors”* [P02, P10, P29], *“the form and clarity with which the lab results are explained”* [P28] and *“the feeling of guilt by the donor”* [P03, P28].

There was a notifier who stated that the best strategy is to overdiagnose: *“I prefer to be told that indeed I am sick so that I continue to have laboratory tests…until a possible disease is completely ruled out, than to be told I am healthy and in twenty years I die from liver cancer because I had hepatitis”* [P03 (3:40)].

The emotions and feelings awakened in the notifiers (during the notification process) had to do with *“sadness because of the state of mind of the donors*”, *“the preoccupation about the situation of the donor”*, *“the disappointment at being unable to handle the emotional state of the donor”* [P02, P03, P08, P09; P25, P28, P38] and *“the anger and ire caused by the reactions of some donors”* [P02, P03, P10, P25]. As explained by one notifier: *“It makes me experience as much…indeed, sadness, sadness when I say ‘sh-t’ perhaps they are persons with little support…; anger, also on some occasions, when the donor tell me his/her sex life includes risky practices or when his/her partner was the person who infected him/her. And…bad vibes, it generates anger and ire”* [P02 (2:87)].

And *“fear because of the donor and what may happen to him/her”* [P25]. The notifier further stated: *“My preoccupation is bigger when they cross the door and leave; are they [refers to physicians who will provide them care at healthcare units] really going to control the risks? Are they going to listen to them? What care are they going to deliver to a person who is not insured, who is not affiliated to popular insurance?”* [P25 (25:13)].

There were some who stated they felt *“indifferent”* and added *“I only look at the donor when I am providing him/her information”* [P01, P29, P30, P31]. One of the reasons was, for example, *“I do not like to notify [silence] because I do not like to be in touch with those emotions, to give that type of news…; yes, indeed, those are minutes in which one even feels badly for them [silence]”* [P30 (30:34)].

Another notifier felt “*amused*” and even stated that *“the very diverse way in which the donors react during notification can drive me to madness”* [P10]. This notifier also said: *“Once a young woman arrived with 50 ELISA tests, all of them negative, but she said that she was infected that there had to be an error, that she was infected…that the labs did not detect her condition…She told me: ‘It is because I work with a guy who has HIV, in a small, closed, cubicle, back to back’. And I thought My God! That is not a risk situation, well, then it could drive me to madness”* [P10 (10:16)].

#### Consequences of tagging the donor with the expectation of a disease

All of the notifiers acknowledge that one of the main consequences of the notification process is the alteration of the donor’s state of mind: *“aggressiveness”*, *“anxiety”*, *“fear”*, *“stress”*, *“confusion”*, *“uncertainty”*, *“loss of hope”*; *“they cry, yell and leave in a state of shock”* [P01, P02, P03, P08, P09, P10, P25, P28, P29, P30, P31, P37, P38].

*“One donor left exploding, saying things, bad-mouthing that he was going to take revenge on someone… At the end of the notification he stated that he felt well but experience itself tells you who is the person that leaves feeling tranquillity and who is the person that will not. The man I have referred to left throwing things and running into people while walking; this running over things and people tells you this person is not well, that he is not well….”* [P09 (9:231)].

*“I have a very special case of a relative that…had a new born daughter who was in the hospital because she had hepatitis. The physicians informed him that she was jaundiced. At that age they could not say that she had hepatitis, not to a new born, then he went home and committed suicide because his daughter suffered from an illness that he had transmitted her….”* [P37 (37:48].

In the same line, many notifiers acknowledged that as a consequence of the notification process, the different areas of the donor’s life can be impacted: with his/her partner, *“infidelity, divorce”*; with family, *“to inform or not what has happened”* and with work, *“discrimination”* [P03, P08, P09, P10, P25, P28, P29, P30, P37, P38].

*“One donor made the following commentary to me: ‘I told them at work that I had Hepatitis C and then I was discriminated… I can no longer go to the bathroom… The entire month I was treated as someone suffering from the pest [nervous laughter]’. And I replied to the donor: ‘You do not even have that diagnosis; that is, you should not have revealed what you did, especially in your workplace if your diagnosis had been not confirmed….’”* [P03 (3:28)]

They also identified physiological impacts: *“They blush, sweat, become pale, with sweaty hands and crack their fingers…”* [P28]. However, one notifier described how to effectively carry out the notification process: “*In order to complete a good notification you even have to work on your own demons. I mirror myself in the people…work on my own demons. I consider the notification process as a violent slaughter. The worst thing that you can do to a donor is to take away the hope that his lab results be negative”* [P10].

#### Barriers and facilitators

The main barrier identified by most of the notifiers was the lack of training as a notifier [P03, P08, P09, P10, P28, P29, P30, P31, P37, P38].

*“Here, no one has taught you anything…it is like a public speaking contest with the donor, doubts will emerge then and you have to explain them to him…”* [P08 (8:62)].

Another important barrier identified by the majority of the notifiers was the mental difficulty of the donors to make a reasonable judgment because of the way they are requested or called to come to the BB to find out their test results. The manner in which they are requested to come depends (they said) on three events: “*the communication means used to make the request (postal, telephonic, or electronic)”*, *“the person who calls them [here, they spoke about personnel rotation] and what they are told during the call”* [P03, P08, P09, P10, P28, P29, P30, P31, P37, P38]. One notifier highlighted: *“The donor arrives to demand attention, he/she comes in a crisis state, they do not listen, what they want is for someone to solve the problem that he/she has; then, they are blocked…do not listen to you…”* [P09 (9:4)].

The *“lack of psychological support for the donors”* was identified as a barrier by several of the notifiers: *“only those with HIV receive it”* [P08, P10, P28, P29, P30, P31]. And *“The short time available to disclose the lab tests’ results and the overpopulation of donors we have to care for”* [P09 (9:46)].

There is also the fact that *“The donors lie in their declarations about their sexual risk factors and personal data for post-donation contact”* [P09, P25, P28, P37].

*“You ask for a phone where he can answer, and then if you call them at home and you are in contact with him; well, there is no better way, right? Sometimes they give you false phone numbers. Right now, we have a donor who reacts to HIV and the phone numbers for the place where he works, his cell phone and home are false… When a person lies, I understand that that person has risk factors, right? that is why he denied all that information…”* [P09 (9:27)].

The identified facilitators were *“Training the notifier on how to disclose to the donor his/her screening test results and how to provide psychological support given their emotional reaction”; “To improve the already standard way of locating reactive donors or even to implement a programme that takes care of the entire process of localisation”; “To improve the manner of the notifier―donor interaction, such as the importance of the first greeting, the volume of the voice and to call them by their name…”* [P02, P08, P10, P25, P28, P30, P38].

### Donors

Except for one donor, whose donation was altruistic [P33], all the others stated that they had made a family/replacement donation.

Three non-medical notifiers, two social workers and an accountant, were the ones who localised the donors with reactive viral marker results. They were localised and requested in the following manner: by telephone call, speaking directly with the donor [P05, P07, P12; P15, P16, P17, P18, P21, P22, P23, P24, P33, P36]; by telephone, through a relative [P04, P06, P11, P13, P19, P26, P27]; postal service [P14, P18, P23, P35] and telegram [P20]. Two donors did not describe how they were requested to come [P32, P34].

#### Beliefs

Several donors emphasised that they considered themselves to be *“healthy and without risks”*, without explaining their potential illnesses [P04, P05, P11, P13, P18, P19, P23, P24, P32, P33, P34]. Many attributed their lab tests’ results to different causes, such as *“the presence of diabetes in the family”*, *“failure to wash hands”*, *“a needle puncture in one finger”*, *“the meals consumed*”, *“lack of sleep”*, *“throat infection”*, *“vertebral column problems”*, *“drug consumption”*, *“alcohol consumption”*, *“use of certain combs or nail clippers”*, *“a previous deferral as a donor”*, and “*presence of bedbugs at home”* [P05, P06, P07, P11, P13, P14, P15, P16, P17, P18, P20, P21, P22, P23, P32, P33, P34, P36].

#### Emotions and feelings caused by the process of notification

The emotions and feelings reported showed the three phases of the notification process: the initial phase, the notification process itself and the post notification phase.

The initial phase refers to the call or request for the donor to come to the BB to know his/her screening test results. The call was made by three non-medical notifiers. Each one of the donors was told that *“something”* had been found in their initial results and that they should come to the BB to receive more information. From then on, the donor did not receive further information until he/she came to the BB and had a face-to-face interview with the notifier to know the test results.

As a result of this call, all of the donors stated that they felt a wide range of emotions and feelings, such as *“surprise”*, *“fear”*, *“fearful amazement”*, *“crying”*, *“anxiety”* (*e.g. “a knot in the throat”*), *“sadness”*, *“nervousness”*, *“stress”*, *“anger”*, *“suffering”* (*e.g. “it was terrible”, “I felt badly”*), *“suspense”*, *“sinking feeling”* (*e.g*. *“strange”, “frustrated”, “doubtful”*),*“rejection”*, *“depression”*, *“thoughtful”*, *“insecurity”*, *“disillusion”*, *“disappointed”*, *“lack of trust”* and *“preoccupied”* [P04, P05, P06, P07, P11, P12, P13, P14, P15, P16, P17, P18, P19, P20, P21, P22, P23, P24, P26, P27, P32, P33, P34, P35, P36].

*“The person who called me on the phone told me: ‘Go to the doctor [he/she is referring to the notifier] there he/she will have a better explanation’. I answered: ‘Good, are you a nurse or a secretary? Or what are you?’ And he/she replied: ‘Don’t get angry…’ I answered: ‘I am not angry, but there should be a person here trained to solve doubts…’ [repeatedly strikes the table with his fist]”* [P11 (11:8)].

*“The biggest fear, that it could be something very serious, and that my life was already limited, that is what I felt”* [P17 (17:9)].

*“Well, badly… [tries not to cry] I really feel like that because they don’t tell me the things [silence again]. I feel as if I had a knot in the throat…I wish they would tell me what I have so that I would be quieter [there is another silence]. The lady physician told me that it was not serious but whether you want to or not you are always doubtful. Why did they not tell me at that moment that I could not donate blood? I do not understand, no, I do not understand”* [P12 (12:14)].

The phase of the notification process itself encompasses the time during which the notifier interviews the donor and discloses the reactive viral marker results. All the donors reported that: “*The physician [the notifier] asked me many questions and did not clarify my doubts”*. They also stated that: *“I did not understand the information received”* [P04, P05, P06, P11, P12, P13, P14, P15, P16, P17, P18, P19, P20, P21, P22, P23, P24, P26, P27, P32, P33, P34, P35, P36]. Several stated having felt *“blocked”* [P06, P12, P16, P23, P34].

*“At the time I thought it was a serious disease, I did not know what to do…For instance, all that day I thought what if I had this? And if I had that other? Will I tell my family? Will I not tell my family?”* [P16 (16:19)].

*“…As being ignored, that is what the lady physician made me feel [he refers to the notifier], as being ignored; that is, as if she were saying: ‘Well you are screwed’. And I thought Ah! C’mon!, as if I wanted to be here…I really prefer, I swear to you, I prefer put money together and spend it in a lab…I think that they made a mistake, they made a mistake, they gave me someone else’s results, that is what my brain is thinking”* [P18 (18:41)].

*“I was not given the test that that I had undergone, and I asked him [the notifier]: ‘What type of test did you use?’ And he replied: ‘It was the rapid diagnosis test’. Then, I answered: ‘Yes, but what type of test did you use? How reliable is it?’ He replied: ‘That is, it is a blood bank!’ He almost told me: ‘Listen stupid little girl! It is a blood bank!’”* [P04 (4:25)].

The emotions and feelings described were *“anger”, “sadness”, “preoccupation”, “depression”, “nervousness”, “fear”, “sacredness”, “lack of confidence” and “deception”* [P04, P11, P12, P14, P17, P18, P19, P22, P24, P33, P34, P35, P36]. One of them even thought of committing suicide if they told him he had AIDS [P33]. Only two donors mentioned trying to be at ease during the notification [P20, P21].

*“Well, right now I did feel, not anger, but ire, not against me, but against the person [refers to the notifier] neophyte, ignoramus, or whatever she is, she is wearing a butcher’s coat although she calls herself doctor…she was unable to offer me an explanation…Now that I am rethinking the whole thing, I see that that she prescribed an option [he refers to new lab tests], I will see what she tell me about the option. If she tell me the same, I will send her to go and screw his mother…as simple as that”* [P11 (11:22)].

The principal motive for being preoccupied felt by all the donors was “*the uncertainty about the type of lab tests they should get done”* to confirm or reject the illness. For many donors, it was also *“the possibility of being sick”* [P04, P05, P06, P11, P14, P15, P17, P20, P21, P22, P23, P27, P33, P35, P36]. In addition, every one of them mentioned being preoccupied about the potential impact on their family.

*“What if during those days I infect my family? …I imagine that disease travels through the blood, Is that true? He even did not even tell me [referring to the notifier] Do you know what? Right now, you must abstain from sexual relations. The only thing that told me was: ‘Alcohol is prohibited’. And what about alcohol? So, he seems to me as I having an alcoholic face or what? The only thing that prohibited me was alcohol…and as far as I know alcohol does not make us ill….”* [P18 (18:40)].

Several donors emphasized the saying about *“Whether they should communicate their family the information received and how to do it”* [P04, P12, P13, P18, P19, P23, P24, P26, P32, P36]. One of them stated: *“What will they say that I am going to bed with everyone in the world”* [P16]. Only four donors indicated in a specific manner their preoccupation about having HIV [P07, P16, P19, P33].

The phase after the notification has to do with what the donor experiences when he/she knows the second results of viral markers. Several of the donors mentioned feeling *“relieved”* and *“released”* [P05, P06, P07, P15, P16, P19, P23, P26, P27, P32, P33]. Among them, one donor stood up because he said he was particularly *“happy!”* because he had been informed that *“it was not HIV”* despite acknowledging he had engaged in risky sexual activities [P07].

Some donors whose second set of viral marker results were indeterminate or reactive reported to have researched on the internet [P05, P06, P18, P19, P20] due to the confusing nature of the information received. Others mentioned that they immediately underwent the tests in a private lab and have gone to another doctor for a second opinion [P04, P13, P20, P23, P34, P35, P36].

#### Consequences of tagging the donor with the expectation of an illness

After the notification of the test results, the donors identified a series of repercussions in the different spheres of their lives: individual sphere, with their sexual partner, with their family and children and with work and social life.

With regard to the individual sphere, all the donors presented, to a greater or lesser degree, a gamut of emotions and feelings, as previously indicated. Some added loss of appetite and concentration, with headaches and insomnia [P12, P15, P17, P18, P26].

*“For me it is indeed very impacting…I do not know what I am going to do; moreover, I do not even have the desire to go to work, I swear, right now I do not want to go to work, but, also, I do not want to go home…Hunger has gone away…I believe I am going to walk for a while, I am going to think, I am going to see what I am going to do… I am having many doubts, many, many doubts. I will think about what to say at home…I do not know if I should eat in the same dishes, if I should use the same spoons, I do not know….”* [P18 (18:38)].

With regard to their sexual partner, several donors stated that they felt *“an absence of sexual appetite”*, *“rejection of intimacy for fear of infecting”* and *“discussions with their partners”* [P06, P07, P18, P19, P22, P23, P27].

With regard to their family, half of the donors mentioned that they distanced themselves from their children for fear of infecting them [P04, P06, P07, P11, P12, P18, P23, P24, P32, P33, P35, P36].

*“… I no longer wanted to get near my children…I saw them this way [he retreats into his seat]. My youngest child would ask me ‘give me a kiss!’ I gave him a kiss reluctantly…I did not reject them, I just put them aside. My oldest boy would ask me: ‘Are you sick?’ I would tell him: ‘No, I am well son.’ Up to there and not more [Sigh].’* [P06 (6:31)]

With regard to work and social life, several donors emphasised the lack of concentration at work, absence from work, being ridiculed in workplace and rejection. There were even some who stated that “it had changed their lives” [P04, P06, P07, P12, P15, P18, P22, P27].

*“Well…imagine that I got to work, and I only wanted to be like this [he retracts into his seat]. Or sometimes wanted someone to come near me and ask How are you? How do you feel? Or something like that, something like that”* [P06 (6:55)].

*“…It is impacting my work a whole lot, in my daily life…; and in my family life, all of a sudden we have a discussion, and those fights remain here, in my head and I take all that to work…indeed it is impacting me a whole lot….”* [P12 (12:36)].

#### The idea of ‘permanent rejection’ or ‘lock’

Half of the donors reported to have understood that permanent deferral meant that they would not be able to donate blood again; nevertheless, they stated that they had not understood the reason for the deferral [P06, P07, P11, P12, P13, P16, P17, P18, P21, P22, P23, P24]. One donor said: *“Because of standard procedures I would not be able to donate blood again; despite the fact that my final lab result was negative”*; notwithstanding, he said: *“I would donate blood again if it were necessary”* [P15]. In contrast, another donor stated that he would not donate blood again because he did not want to know anything about BBs [P04]. Several donors did not understand whether they were permanently deferred for future blood donations [P05, P14, P19, P20, P26, P27, P32, P33, P34, P35, P36].

#### Barriers and facilitators

The barriers more frequently mentioned referred to the notifier–donor relationship. Several donors described the notifier as a *“cut to the bone”*, *“cutting off”* person; someone who closes off access to the options: *“he does not allow me to talk and only refers me to other physicians”* and *“he does not clarify doubts”* [P04, P07, P18, P19, P20, P23, P24, P32, P33]. They also identified the short time for receiving care as a barrier: *“between seven and fifteen minutes”* [P04, P07, P15, P16, P18, P24, P32, P33].

*“They told me in the blood bank that I had hepatitis C, they never corroborated their statement, they sent me to the family physician… A terrible situation! Because he was never able [he/she is referring to the notifier] to tell me what happened. I told him: ‘But how is it that you are going to send me to my family physician?’. To which he added: ‘Yes you are going to go…your lab test is reactive’. And I told him: ‘But why? Why don’t you corroborate?’ He insisted: ‘No, you go with your family physician’. That way, cutting me off….”* [P04 (4:11)].

Some identified as a barrier the fact of *“not remembering anything”* of the information given to them during the notification. This was described as *“it was erased from my mind”*, *“out of sync”*, *“the brain works slowly”* and *“disoriented because of so many doubts”* [P06, P12, P16, P23, P24].

*“I felt…I felt out of sync! … nervous, it erased from my mind …; that is, I only intended to find out the diagnosis of what I felt, that is, I went only to find out what I had nothing more….”* [P06 (6:28)].

Among the facilitators stood out the fact that the news (from the call in the initial phase) should have been communicated *“not as abruptly” “in a personal manner and not to the relatives”* [P16, P18, P19, P22, P24, P27, P33].

*“… I would have liked for them to call me on my mobile phone, not my mother so as not to preoccupy her…that I would be the only one to find out and not involve my mother because everyone is scared”* [P22 (22:45)].

*“They should look for…some way to tranquillise you by phone, and say You know what? That we could not do the tests, or your results are lost for some reason…; with technical but general terms…it is not the same ‘aw, what happens is that it could be that you have something’”* [P27 (27:11)].

Likewise, during the notification process there were facilitators, such as: *“a more human treatment from the notifiers”*, *“with tenacity”*, *“with comprehension”*, *“with compassion”* and *“with empathy”* [P04, P07]. Also, *“psychological support”* and *“a longer time for the notification”* [P04, P12, P16, P18, P33]. One donor emphasised the need for the notifier to be trained [P19].

## Discussion

This paper presents the methods and findings of a study based on individual, face-to-face, semi-structured, in depth interviews involving notifiers and blood donors involved in the notification process of permanent deferral, from the perspective of the notifier-donor relationship. Thematic analysis yielded eight themes related to the participating subjects’ perceptions and practices concerning notification process of permanent deferral. Informants spoke about their values, beliefs, feelings, interests, anxieties, concerns, and their lived experience. Although an oft-referenced limitation of qualitative research is its lack of generalizability (discussed further below), the insight gained through this study would not have been captured using standard quantitative survey.

A series of complex, interrelated findings were identified. The most significant related to the way notifiers disclose information. They acknowledged that their obligations are to provide (the donors) the viral marker results and their medical meaning, to inform about the deferral status in relation to future blood donations, and to make clear recommendations about the course of events to be followed.

The findings also indicated that the notifiers believe that the fact of disclosing positive or reactive or indeterminate results and, consequently, tagging the donors as permanently deferred reduces their capacity to understand and make a reasonable judgment. Therefore, they would prefer to be brief and clear in terms of the type and amount of information they communicate to the donor.

This study also revealed that when notifying results that are ‘reactive’ or ‘indeterminate’, they are communicated by the notifiers to the donors using words that mean that they will no longer be able to donate blood in the future, even if the results of the confirming diagnosis were negative.

These attitudes and practices of the notifiers reflect what is legally required, that is, what the Mexican Official Standard for the Disposition of Human Blood has stated in this respect (NOM-253-SSA1-2012; subsection 5.2.4 and all items included in subsection 6) [3].

Another finding was that almost all of the notifiers continued this practice despite acknowledging, by themselves, the psychological and social risks to which the donors are exposed when they are informed about the diagnostic uncertainty and the consequent permanent deferral. The set of side effects (mental, social, etc.), which are derived from the process of notification, described by the participating donors are consistent with the reports of other authors [8–12].

The ethical problem caused by these attitudes and practices emerges when the notifiers base their judgements and practices only on the positive obligations (e.g., guidelines and standards) of the beneficence they owe to the donors. This problem provides several points that warrant discussion.

First, the notifier―donor relationship is linked to a specific function in the provision of care in public health [19]. When a donor comes to a BB, the BB acquires the obligation of providing beneficent care (through its health professionals); this obligation is associated with its function. The salient point here is the fiduciary nature [13] of the relationship established between the BB and the donor. The fiduciary duty is an essential protection in notifier-donor relationships. This is why the obligation of the notifier to provide beneficent care is wrongly interpreted if it is defined and implemented based only on what is stated in the Mexican Official Standard for the Disposition of Human Blood [3].

Second, although the goal of BBs is to ensure blood safety and availability to meet the transfusion needs of each and all patients; it also must be concerned with the promotion of the well-being of the donors, for donors are as human as a patient and as such they should expect that their own right to beneficence and non-maleficence will be respected. This fact is the fundamental reason of the moral justification of the donation. Preventive medicine and all active interventions of public health have been adopting social actions of beneficence, such as health education. This has to do not only with physical functioning but also with working toward achieving the wholeness of individuals and the specific populations to which they belong. This includes the obligation to educate the deferred donors on the importance of the medical–epidemiological follow-up to rule out a possible disease, to gain access to treatment as appropriate and to ensure that a stressed donor does not become a victim of diagnostic uncertainty and/or tagged with a potential illness.

Third, the notifiers accuse the donors of irrationality because they (the donors) assume voluntarily greater psychological and social risks; yet, the notifiers do not consider that the perceptions of damage and benefice of humans, in this case the donors, are idiosyncratic and depend on the life story of each, how they perceive themselves and the emotions and feelings awakened in them before their permanent deferral and a potential disease are disclosed.

Donors are put in a situation in which they go from considering themselves as being *“healthy”* to suddenly expecting that they have *“something”*, that is, a probable disease. In that respect, Beauchamp and Childress [13], Laín [20]**,** and Campbell [21], among others, have underlined that in the health professional―client relationship, it is important to have a continuous and frank dialogue that guarantees a clear comprehension of the relevant information being communicated and facilitates its co-execution by the participating agents. The dialogue must allow the notifier to understand the personal and social realities of the donor and allow the donor to understand what is happening, what is possible and what is not possible. This is why ethics is process-based and dialogical, rather than ruled-based.

Fourth, the fact that the Mexican Official Standard for the Disposition of Human Blood only indicates the obligation of notifying and to do it through counselling, without defining how to do it (NOM-253-SSA1-2012. Subsection 5.2.4) [3], does not mean that the notification itself must be considered as an opportunity for the notifier to act based exclusively on his/her beliefs, feelings and emotions and his/her empathy (greater or lesser) toward the deferred blood donor, rather it must be considered for the notifier to use his/her best professional judgment.

The actions of the notifier have a high probability of preventing damage to the health of the donor and suppressing the conditions that could damage the health of others; for example, sexual partners, children and family. This public health assumption implies the moral obligation of the notifier to alert the sexual partners of those deferred donors who refuse to disclose their condition and also refuse to practice safe sex. Only a very narrow understanding of the ethical principles of autonomy and beneficence would support the idea that the notifier has no obligation to provide health care that includes both the individual health of the donor and the health of his/her community.

Most of the notifiers identified the main barriers to the notification process as follows: (a) their lack of training on how to inform donors of the reactive viral marker results; (b) the way in which the donors are ‘called’ or ‘given appointment’ to find out the results (the current way of doing it generates a set of negative emotions and feelings among the donors that directly impact their actions the moment they are informed of their lab results and tagged as permanently deferred); and (c) the short time available to communicate the results to the donors along with the large population of donors served in one day.

The notifiers acknowledged the impact (on the donors) of the disclosure of the test results (positive, reactive, or indeterminate) and information of a possible disease and tagging as permanently deferred. Thus, they identified the following needs: psychological support for all the deferred donors and to improve the quality of the notifier—donor relationship. These needs are consistent with those identified by the participating donors, who added the need of the notifiers to be trained.

It is important to mention that there was a difference between the values of the notifiers and the donors. The notifiers valued compliance with the standard [3] and the donors’ acceptance and adherence to their indications. In contrast, the donors valued honesty during the communication, clarity in the information provided to them and a greater involvement of the notifier in the relationship.

The attributes valued and demanded by the donors belong to the principles of beneficence and non-maleficence [13] and serve as the points of reference to understand what the donors tend to value. Awareness of the disparity of the values at play prevents potential consequences in the notifier―donor relationship, such as resentments toward a notifier who is indifferent and distant, poor communication, lack of satisfaction with the care received and non-adherence to the medical indications.

### Limitations and strengths

The participating BBs belong to the public sector of medical care; therefore, it is possible that the results of this study are not applicable to the private sector. Nevertheless, it is important to emphasise that the Central Blood Bank of the National Medical Centre ‘La Raza’ of the Mexican Institute of Social Security is the second largest blood bank in Latin America [7]. On average, it serves 106,000 donation candidates per year, with 66,300 being accepted and 39,000 being deferred (HEXA-BANK database).

The information generated by this study was gathered through semi-structured, in-depth interviews with the main agents participating in the notification process. This may be perceived as a limitation to understanding the *emic* perspective of Mexican culture since we did not include more ethnographic techniques for the generation of multiple sources of data. Nevertheless, the fact that (a) the participating notifiers differed in terms of gender, age and work experience; (b) the donors differed in terms of gender, age, educational level and potential diagnoses and (c) the participating BBs are national reference centres for several regions in Mexico, providing a solid base to develop a better understanding of the way the notification process of permanent deferral takes place.

During the lengthy recruitment period only one volunteer donor was identified. This provides a snapshot of the real life of Mexican BBs. Although, it is officially reported that only 3% of donors are volunteers, the clinical experience of a haematologist (the first author) speaks that in reality nearly 100% of blood donations occur as family/replacement donations. World Health Assembly resolution WHA63.12 [22] urges all Member States to develop national blood systems based on unpaid donations and truly voluntary; this would keep individuals with risky behaviours from donating and hiding their risky behaviours, or feeling forced to donate.

### What next?

The empirical lessons gained from this study can feed back upon and influence the existing normative requirements (what should and ought to be done) for BBs. Normative ethics is a form of inquire that attempts to answer―in a systematic, reasoned and critical fashion―questions such as: should the blood banking system be responsible for conducting confirmatory testing before communicating unconfirmed screening results and before permanent deferral?

The Mexican official standards [3] stipulates that BBs are obliged to assure a quality screening of all donated blood for transfusion-transmissible infectious. They are also obliged to inform reactive or positive donors of their deferral status and the strategy that must be followed; likewise, notifier-donor relationship is not considered as a doctor-patient relationship [1–3], these are the reason why BBs are not obligated to do the epidemiological follow-up of donors with reactive or positive results of the screening tests. Anthropological and sociological studies can raise questions about the universality of these normative claims.

This study also generates new material for a normative inquiry that attempts to develop specific policies and an action plan—based on evidence—that will enable the development of standardized/nationally available training for those communicating the results. The policies should/ought to be consistent with the idea of reorganizing the functions of the Mexican blood banking system—for the sake of blood donors and to improve the health of the communities the BBs serve.

## Conclusion

The knowledge generated in this study has value because as it allows us to understand how the notification process of permanent deferral is understood and conducted by its two principal agents, especially when the information processing implies serious consequences for the donor and his/her family. Moreover, it provides evidence of the need for specific training to incorporate ethics into the practice of the notifier and thereby transform the notifier―donor relationship from being merely bureaucratic and focused on meeting an institutional commitment into one that is truly professional and is at the service of the donors.

## Supplementary Information

**Additional file 1. **Outline of the topic guide.

## Data Availability

Additional file 1. Outline of the topic guide. The dataset used and/or analysed during the current study are available from the corresponding author upon reasonable request.

## Abbreviations

BB: Blood Bank

## References

[1] Food and Drug Administration. DHHS 21 CFR Parts 606 and 630 [Docket No. 98N-0607] General Requirements for Blood. Blood Components, and Blood Derivates; Donor Notification. 2001. https://www.govinfo.gov/content/pkg/FR-2001-06-11/pdf/01-14409.pdf. Accessed 28 Jan 2021.

[2] Council of Europe Resolutions, Recommendations and Convention. Blood and Blood Components. France: European Directorate for the Quality of Medicines and HealthCare. 2012. www.edqm.eu Accessed 28 Jan 2021.

[3] Estados Unidos Mexicanos. Secretaría de Salud. Norma Oficial Mexicana NOM 253-SSA1-2012, Para la Disposición de Sangre Humana y Sus Componentes Con Fines Terapéuticos. Diario Oficial. 2012. http://www.cnts.salud.gob.mx/descargas/NOM-253-SSA1-2012.pdf. Accessed 28 Jan 2021.

[4] Estados Unidos Mexicanos. Secretaría de Salud. Centro Nacional de la Transfusión Sanguínea. 2021. http://cnts.salud.gob.mx Accessed 28 Jan 2021.

[5] Estados Unidos Mexicanos. Secretaría de Salud. Comisión Federal para la Protección contra Riesgos Sanitarios. 2021. https://www.gob.mx/cofepris Accessed 28 Jan 2021.

[6] Estados Unidos Mexicanos. Secretaría de Salud. Comisión Coordinadora de Institutos Nacionales de Salud y Hospitales de Alta Especialidad. Programa de Acción Específico: Seguridad de la Sangre y de las Células Troncales. Programa Sectorial de Salud 2013–2018. 2015. https://www.gob.mx/cms/uploads/attachment/file/120117/PAE2013_2018.pdf. Accessed 25 Feb 2021.

[7] Organización Panamericana de la Salud. Suministro de Sangre Para Transfusiones En Los Países de América Latina y el Caribe. Washington, D.C. OPS; 2016-2017. 2020. https://iris.paho.org/bitstream/handle/10665.2/52150/9789275321720_spa.pdf?sequ%E2%80%A6 Accessed 28 Jan 2021.

[8] Kiely P, Hoad V, Wood EM (2018). False positive viral marker results in blood donors and their unintended consequences. Vox Sang.

[9] Kleinman S, Wang B, Wu Y, Glynn SA, Williams A, Nass C, Ownby H, Busch M (2004). The donor notification process from the donor’s perspective. Transfusion.

[10] Tynell E, Norda R, Ekermo B, Sanner M, Anderson S, Bjorkman A (2007). False-reactive microbiologic screening test results in Swedish blood donors—How big is the problem? A survey among blood centers and deferred donors. Transfusion.

[11] Whittaker S, Carter N, Arnold E, Shehata N, Webert KE, DiStefano L, Heddle N (2008). Understanding the meaning of permanent deferral for blood donors. Transfusion.

[12] Dodd RY, Stramer S (2000). Indeterminate results in blood donor testing: What you don’t know can hurt you. Transfus Med Rev.

[13] Beauchamp TL, Childress JF (2001). Principles of biomedical ethics.

[14] Bryman A (2016). Social research methods.

[15] Sandelowski M (1995). Sample size in qualitative research. Res Nurs Health.

[16] Kuzel AJ, Crabtree BG, Miller WL (1992). Sampling in qualitative inquiry. Doing qualitative research.

[17] Glaser BG, Strauss AL (2012). The discovery of grounded theory: Strategies for qualitative research.

[18] Miles MB, Huberman AM (1994). Qualitative data analysis.

[19] World Medical Association. Public Health. 2021. https://www.wma.net/es/que-hacemos/salud-publica/ Accessed 28 Jan 2021.

[20] La L-E (2003). Relación Médico-Enfermo: Historia y Teoría.

[21] Campbell AV (1984). Moderated love.

[22] World Medical Association. Blood safety and availability. 2020. https://www.who.int/news-room/fact-sheets/detail/blood-safety-and-availability Accesed 21 Dec 2021.

