# Supplementary material for: Donor notification of permanent deferral: a qualitative study on the perceptions and practices of notifier and blood donor in Mexico
Source: BMC Health Serv Res. 2022 Jun 10;22:761. doi: 10.1186/s12913-022-08103-1 (PMC9185984; doi:10.1186/s12913-022-08103-1)
Supplement: Supplementary file 1 — Additional file 1. Outline of the topic guide. [file 12913_2022_8103_MOESM1_ESM.pdf]

Appendix 1: Outline of the topic guide

The following is a topic guide used to prompt participants to openly convey their viewpoints regarding the permanently deferred blood donors and the notifiers. It was not intended that each of the following questions be asked word by word, but rather that they be used as a memory aid to ensure that all important areas would be covered.

Start interview for all participants

Introduction of the interviewer, the study, and its purpose.

Estimated duration of the interview.

Consent and confidentiality: written consent, audio recording, how the data will be used.

Possibility to stop at any time, or decline to answer any questions without any consequences.

Background for all participants

Demographic data

Interviews with notifiers

For all participating notifiers, the interview started with the question: can you tell me about your clinical experience about the notification of permanent deferral to blood donors?

Responses were usually lengthy. When necessary, prompts were used to cover the following points:

- How do you carry out the disclosure of indeterminate test results? Do you find that the amount of information you present is just the right amount of information?
- Do you involve donors in the decision about their health?
- How do you think about blood donors with indeterminate or positive test results?
- During the deferral notification, when you realize a donor is not going to get any better, what do you do?
- What do you usually do when you suspect that a blood donor is a carrier of a communicable infectious disease? To whom do you inform?
- What do you do that permanent deferred donors carry out your medical indications?

- What aspects of context, attitudes, and experience, facilitate or constrain the permanent deferral notification process?

Interviews with permanently deferred donors

For all participating donors, the interview started with the question: I would like you to tell me about the information you received from the Blood Bank. What do you know about it? Responses were usually lengthy. When necessary, prompts were used to cover the following points:

- How did you learn about what is happening?
- How did you learn about your current health status?
- Did you find that the amount of received information was just the right amount of information?
- How much did all the received information improve your understanding of your health status and of medical course of action to be followed?
- Are you satisfied/comfortable with the decision made?
- How much were you involved in the decision making?
- Did you speak (or have you spoken) to your family about what is happening?
- How do you see yourself in the near future?
